# Supplementary material for: A secreted helminth microRNA suppresses gastrointestinal cell differentiation required for innate immunity
Source: Front Immunol. 2025 Mar 27;16:1558132. doi: 10.3389/fimmu.2025.1558132 (PMC11983496; doi:10.3389/fimmu.2025.1558132)
Supplement: Supplementary Figure 1 — Reference gene selection for RT-qPCR data normalization by BestKeeper and NormFinder. Ovine Actin (ACTB) and mouse Glyceraldehyde 3-phosphate dehydrogenase (GAPDH) were the genes that showed a cycle threshold (Ct) standard deviation ≤0.5 with a coefficient of correlation ~1 according to BestKeeper analysis, and a low stability value according to NormFinder analysis (minimal expression variability). [file DataSheet1.pdf]

Ovine abomasal organoid mRNA

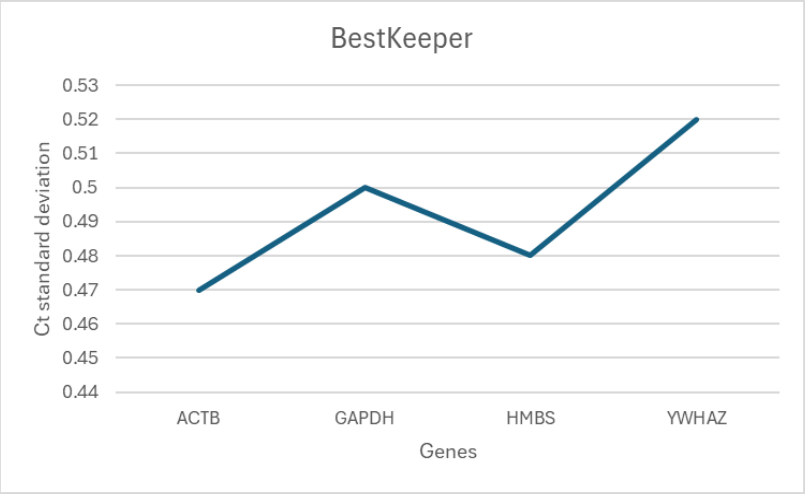

BestKeeper vs.	ACTB	GAPDH	HMBS	YWHAZ
Ct standard deviation	0.47	0.5	0.48	0.52
Coeff. of cor.[r]	0.947	0.91	0.687	0.622
p-value	0.001	0.0024	0.002	0.0041

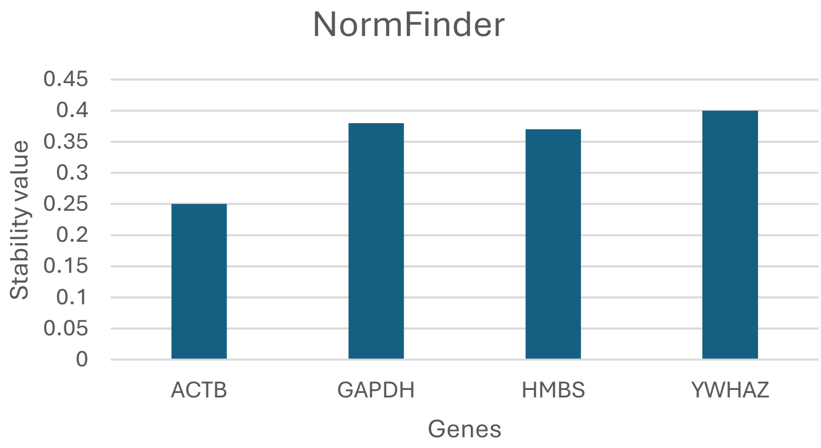

NormFinder	ACTB	GAPDH	HMBS	YWHAZ
Stability value	0.25	0.38	0.37	0.4

Murine SI organoid mRNA

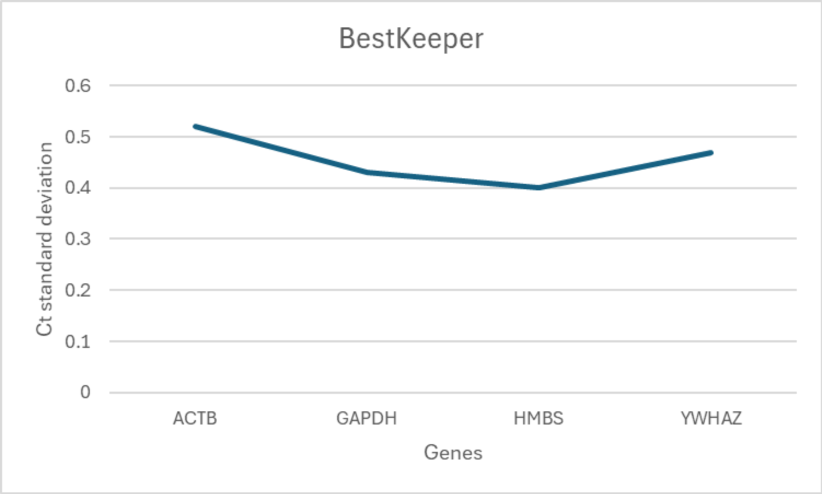

BestKeeper vs.	ACTB	GAPDH	HMBS	YWHAZ
Ct standard deviation	0.52	0.43	0.4	0.47
Coeff. of cor.[r]	0.897	0.947	0.784	0.874
p-value	0.05	0.0017	0.0047	0.0031

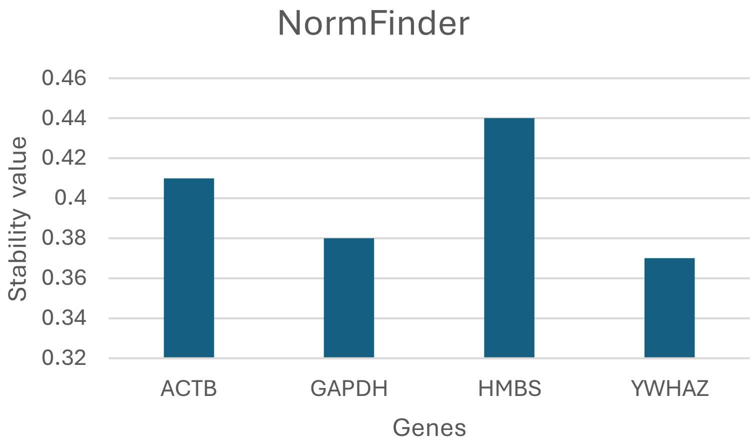

NormFinder	ACTB	GAPDH	HMBS	YWHAZ
Stability value	0.41	0.38	0.44	0.37
