# Supplementary figures and images for: A secreted helminth microRNA suppresses gastrointestinal cell differentiation required for innate immunity
Source: Front Immunol. 2025 Mar 27;16:1558132. doi: 10.3389/fimmu.2025.1558132 (PMC11983496; doi:10.3389/fimmu.2025.1558132)

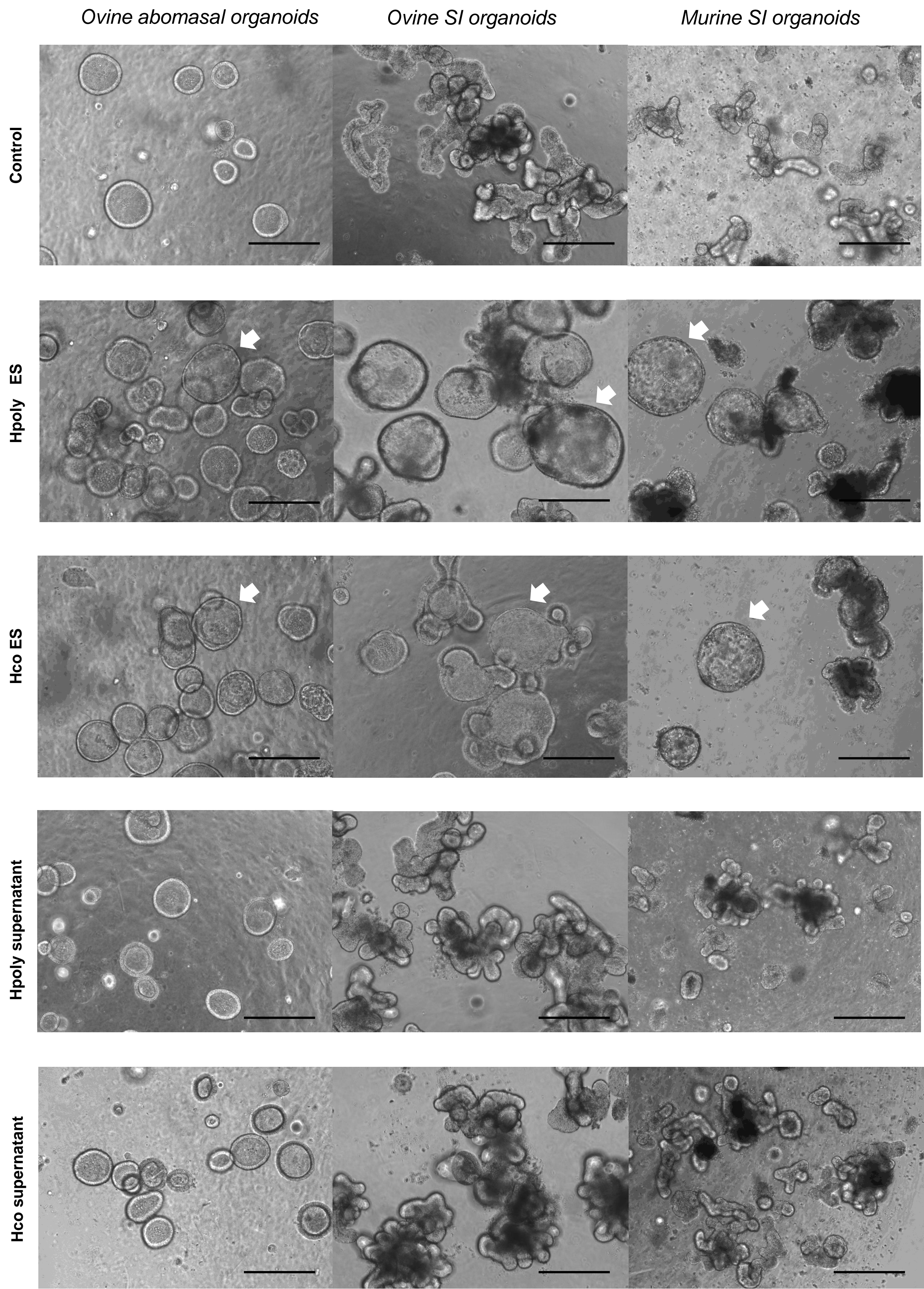

Supplement: Supplementary Figure 2 — Spheroid phenotype in GI organoids after H. contortus or H. polygyrus concentrated ES exposure for 12 h. Representative light microscopy images of ovine abomasal organoids, ovine small intestinal (SI) organoids, and murine SI organoids exposed to concentrated H. polygyrus (Hpoly) and or H. contortus (Hco) adult excretory-secretory (ES) products (10 µg/ml). Control shows organoids in growth medium alone and lower panels show organoids exposed to the flow-through of Hpoly or Hco ES after concentration (3kDa cut-off). White arrows indicate spheroid organoids identified by thinner membrane and more granular appearance. Scale bar = 300 µm. [file DataSheet2.pdf]

A

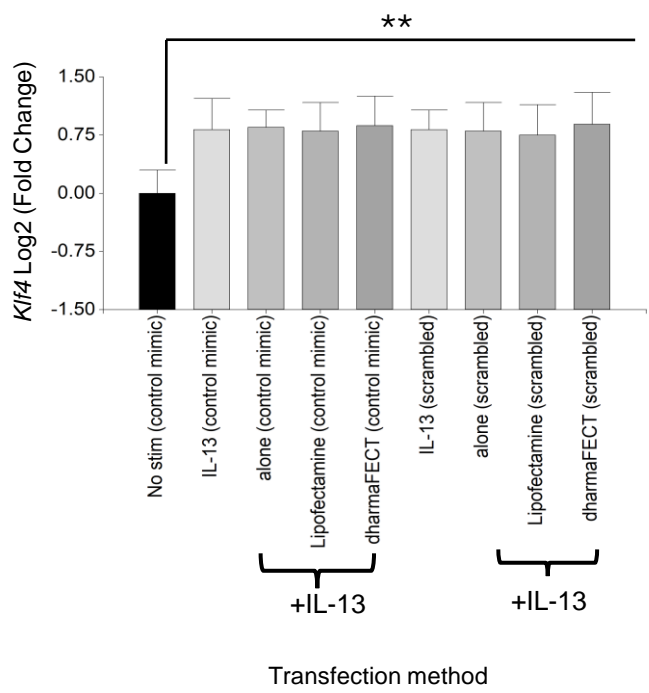

B

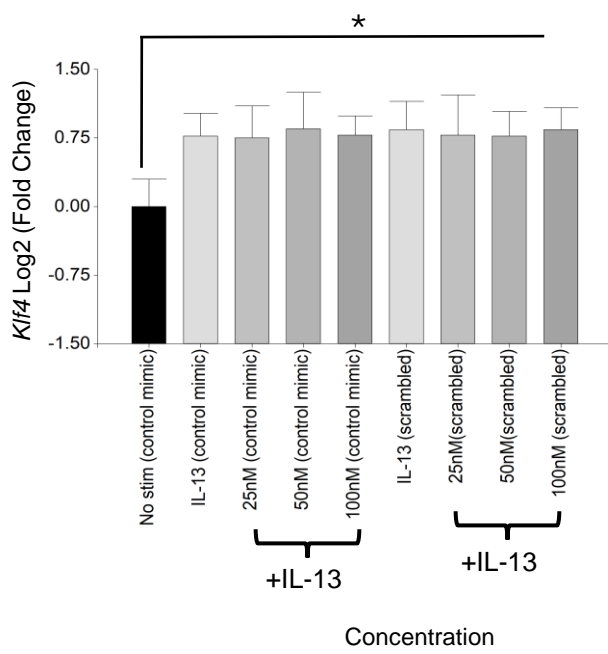

C

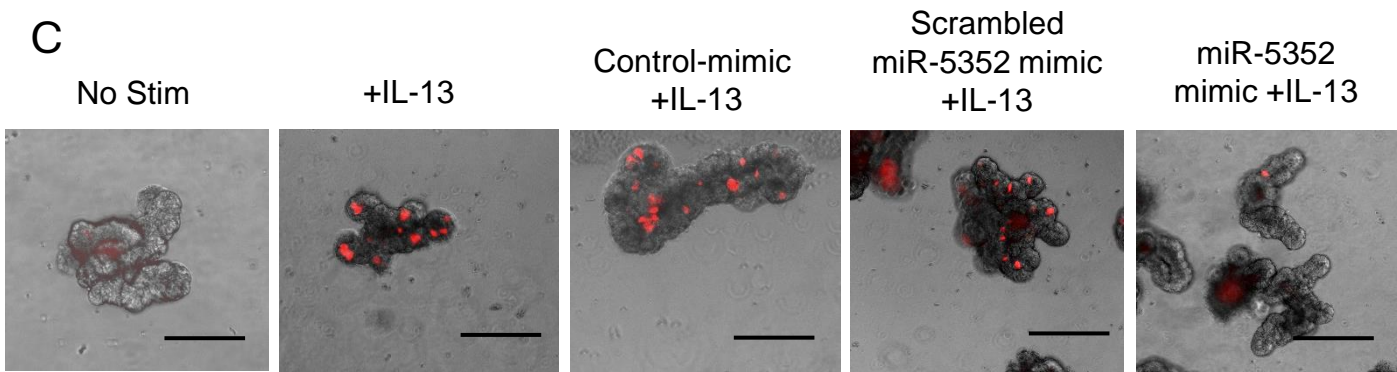

D

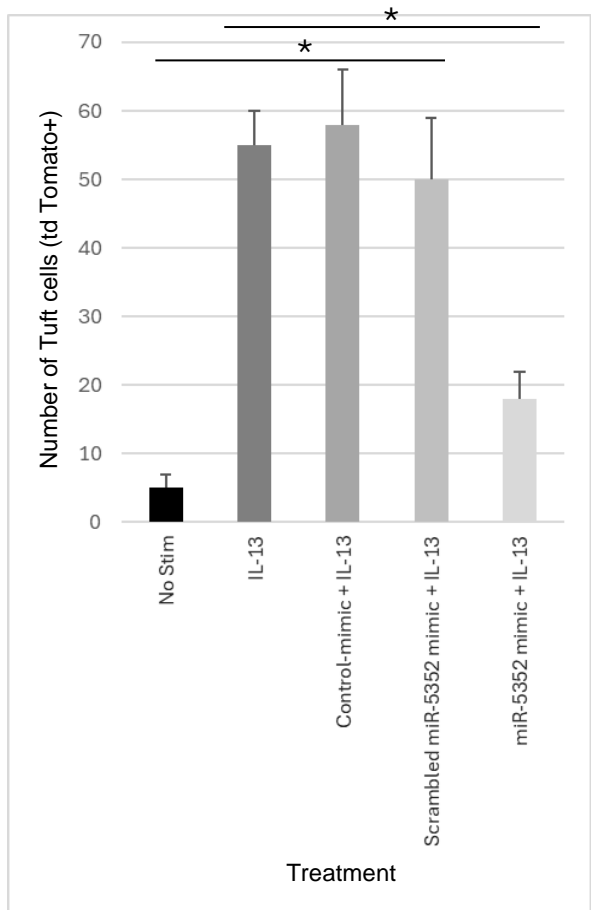

Supplement: Supplementary Figure 3 — (A) Expression of predicted miR-5352 target gene Klf4 in ovine abomasal organoids with no stimulation, treated with IL-13 alone, control mimic (miRIDIAN negative control sequence) + IL13 (alone, with Lipofectamine, or DharmaFECT reagent), or scrambled miR-5352 mimic + IL-13 (alone, with Lipofectamine, or DharmaFECT reagent). miRNA mimics at 100 nM final conc. (B) Expression of Klf4 in ovine abomasal organoids with no stimulation, treated with IL-13 alone, control mimic (miRIDIAN negative control sequence) + IL-13 or scrambled miR-5352 mimic + IL-13, using DharmaFECT as transfection reagent and miRNA mimic at 25, 50, 100 nM. Log2 fold change of RT-qPCR values were compared with non-stimulated control in three independent biological replicates +/- SD. Statistical analysis was by ordinary one-way ANOVA with Tukey’s multiple comparisons test; **, P < 0.01. (C) Representative images of Dclk1 tdTomato+ tuft cells in murine SI organoids, untreated (No stim), treatment with IL-13, control mimic (miRIDIAN negative control sequence) + IL-13, scrambled miR-5352 mimic + IL-13, and miR-5352 mimic + IL-13, using DharmaFECT as transfection reagent and mimics at 50 nM in OGM for 4 days at 37°C. (D) Number of Dclk1 tdTomato+ cells in murine SI organoids at 4 days after treatment with IL-13, control mimic + IL-13, scrambled miR-5352 mimic + IL-13, and miR-5352 mimic + IL-13, using DharmaFECT as transfection reagent and mimics at 50 nM. Graph shows the average number of tdTomato+ cells from 5 images (250 organoids per treatment). Statistics calculated with a two-tailed t-test on mean of biological replicates (n=2) compared to untreated organoids. Scale bars: 100 µm. [file DataSheet3.pdf]

A

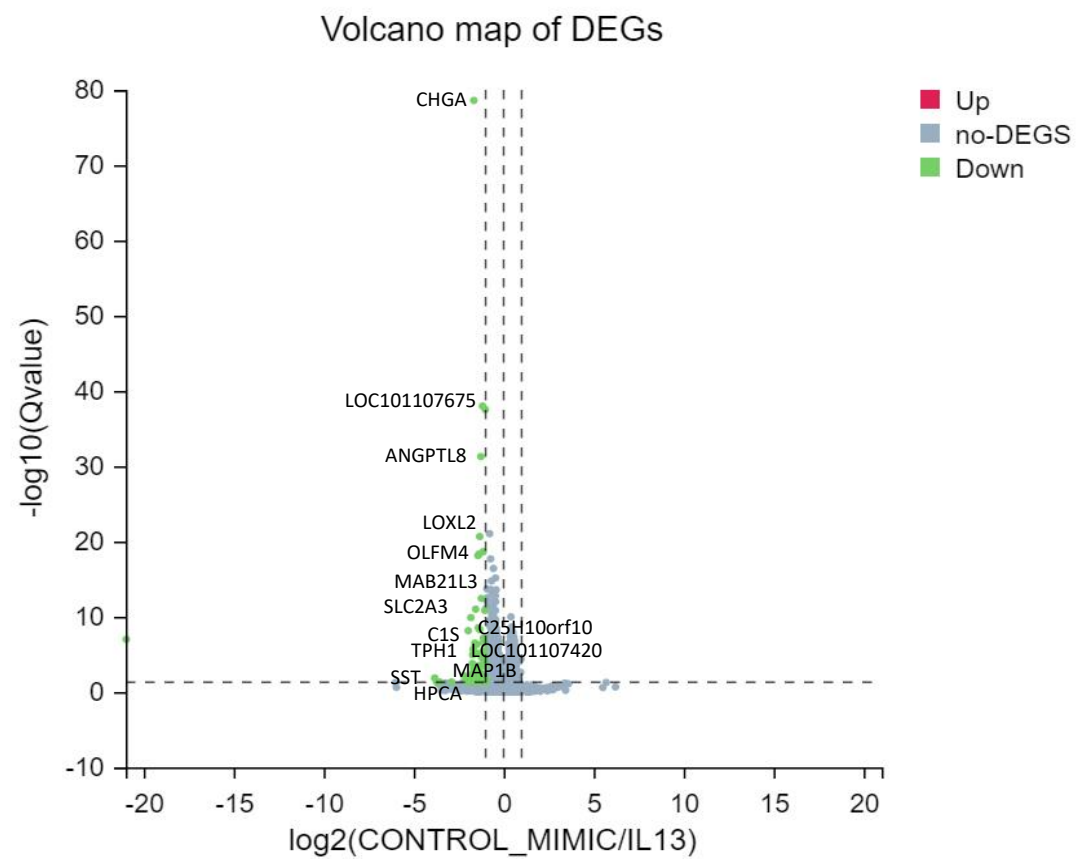

B

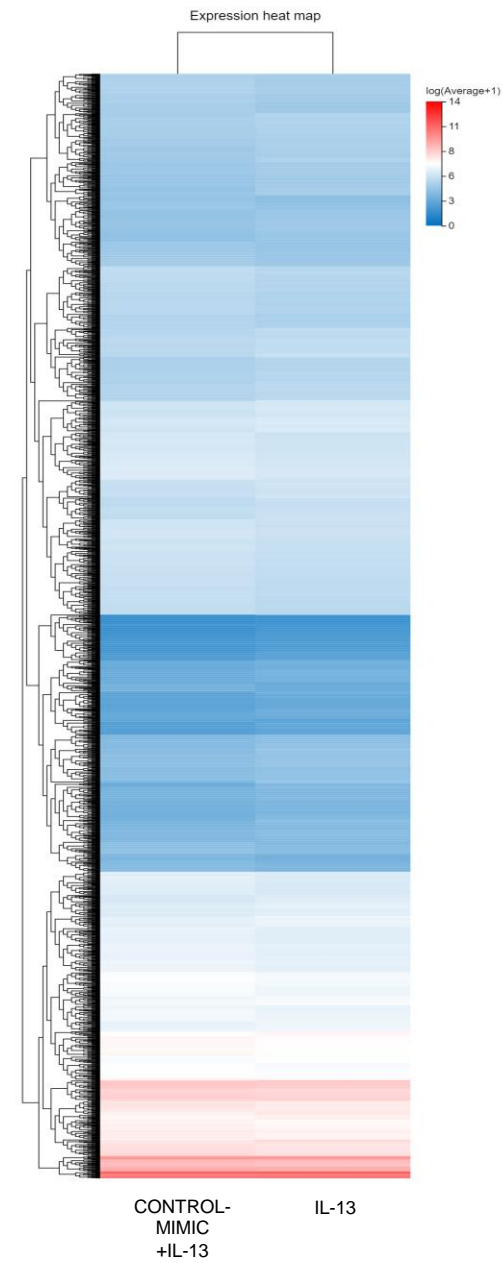

Supplement: Supplementary Figure 4 — (A) Volcano plot of differential expression analysis (DESeq) in abomasal organoids treated with IL-13 plus control mimic vs. those treated with IL-13 alone and cultured for 48 h. x-axis represents the fold change of the difference after conversion to log2; y-axis represents the significance value after conversion to -log10. (B) Heat map showing expression level of genes from ovine abomasal organoids treated with IL-13 plus control mimic and IL-13 alone using normalised average read counts, standardized method: log (value+1). Colours indicate level of expression from low (blue) to high (red). [file DataSheet4.pdf]
